# Pathogenic *PSEN1* Thr119Ile Mutation in Two Korean Patients with Early-Onset Alzheimer’s Disease

**DOI:** 10.3390/diagnostics10060405

**Published:** 2020-06-14

**Authors:** Eva Bagyinszky, Hyon Lee, Jung Min Pyun, Jeewon Suh, Min Ju Kang, Van Giau Vo, Seong Soo A. An, Kee Hyung Park, SangYun Kim

**Affiliations:** 1 Department of Industrial and Environmental Engineering, Graduate School of Environment Gachon University, Seongnam 13120, Korea; navigator120@gmail.com; 2 Department of Neurology, Gachon University Gil Medical Center, Incheon 21565, Korea; hyonlee@gmail.com; 3 Department of Neurology, Seoul National University College of Medicine & Neurocognitive Behavior Center, Seoul National University Bundang Hospital, Seongnam 13620, Korea; pyun.jungmin5@gmail.com (J.M.P.); viest43@hotmail.com (J.S.); 4 Department of Neurology, Veterans Medical Research Institute, Veterans Health Service Medical Center, Seoul 05368, Korea; minju0321@naver.com; 5 Institute of Research and Development, Duy Tan University, Danang 550000, Vietnam; giauvvo@gmail.com; 6 Department of Bionano Technology, Gachon University, Seongnam 13120, Korea; seong.an@gmail.com

**Keywords:** Alzheimer’s disease, Thr119Ile mutation, presenilin-1, next generation sequencing

## Abstract

We report a probable pathogenic Thr119Ile mutation in presenilin-1 (*PSEN1*) in two unrelated Korean patients, diagnosed with early onset Alzheimer’s disease (EOAD). The first patient presented with memory decline when she was 64 years old. Magnetic resonance imaging (MRI) scans showed diffuse atrophy in the fronto-parietal regions. In addition, 18F-fludeoxyglucose positron emission tomography (FDG-PET) showed reduced tracer uptake in the parietal and temporal cortices, bilaterally. The second patient developed memory dysfunction at the age of 49, and his mother was also affected. Amyloid positron emission tomography (PET) was positive, but MRI scans did not reveal any atrophy. Targeted NGS and Sanger sequencing identified a heterozygous C to T exchange in *PSEN1* exon 5 (c.356C>T), resulting in a p.Thr119Ile mutation. The mutation is located in the conserved HL-I loop, where several Alzheimer’s disease (AD) related mutations have been described. Structure analyses suggested that Thr119Ile mutation may result in a significant change inside conservative loop. Additional in vitro studies are needed to estimate the role of the *PSEN1* Thr119Ile in AD disease progression.

## 1. Introduction

Early-onset Alzheimer’s disease (EOAD) represents a minority of all Alzheimer’s disease (AD) cases (1–6%). The early onset form of disease occurs between 30 and 65 years. EOAD-associated mutations with high penetrance are even rarer, with the frequency of the amyloid precursor protein (*APP*), presenilin 1 (*PSEN1*), and presenilin 2 (*PSEN2*) accounting for 1%, 6%, and 1% of patients with EOAD, respectively [1,2,3,4]. Mutations in *PSEN1* have been described the most frequently, and more than 300 pathogenic mutations have been summarized in the Alzforum database (www.alzforum.org/mutations). *PSEN1,* which encodes the presenilin-1 protein, constitutes the catalytic subunit of γ-secretase complex [5,6]. Specific mutations in the *PSEN1* gene may impair γ-secretase’s function to cleave APP, resulting in higher Aβ42 and/or lower Aβ40 levels [7]. Majority of patients with *PSEN1* mutations usually develop an AD phenotype at the age of 40–60 years. Additional *PSEN1* mutations have been associated with young onset, where patients developed neurodegenerative symptoms in their 30s or earlier [8,9,10].

In this study, we report a probable pathogenic *PSEN1* mutation, Thr119Ile, in two unrelated Korean patients. This mutation was discovered in an Argentinian family for the first time, where the affected family members presented with a similar disease course at a similar age of onset. This is the first time this mutation has been reported in the Asian population. One of the patients with Thr119Ile was published in a research article in 2019 by our group, where 67 Korean EOAD patients went through next generation sequencing analysis [11]. In this manuscript, two detailed case reports were introduced with detailed clinical symptoms and structural predictions were also performed on the mutation to assess its pathogenicity.

## 2. Materials and Methods

### 2.1. Clinical Findings

**Figure 1:**
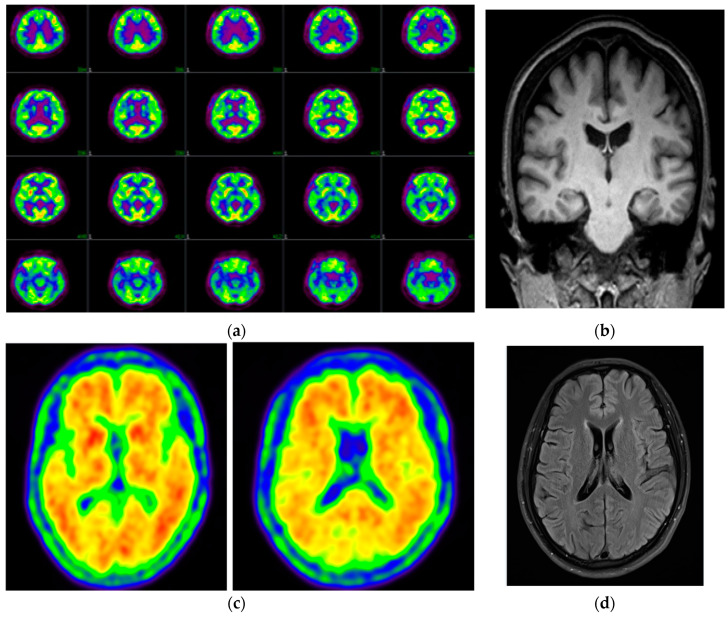
(**a**) 18F-fludeoxyglucose positron emission tomography (FDG-PET) of patient 1 showed reduced metabolism in different brain areas (bilateral temporal and parietal cortices). (**b**) Magnetic resonance imaging (MRI) imaging data of patient 1 revealed atrophy in bilateral parietal cortices. (**c**) PiB-PET data of patient 2 revealed amyloid positivity in several brain regions (bilateral lateral temporal-frontal-parietal areas). (**d**) MRI imaging data of patient 2 did not reveal any atrophy.

The first patient was a female, who presented memory dysfunctions at 60 years of age [11,12]. Patient was working as a schoolteacher, and although she did not experience any difficulty teaching, she struggled in planning courses. In addition, she could not remember conversations she had with her colleges a few days prior, and became slower in completing her tasks. Later, her symptoms progressed and language impairments, such as word-finding, difficulty became apparent. Her clinical dementia rating (CDR) score was 0.5/3, and her mini mental status exam (MMSE) score was 24/30. FDG-PET revealed reduced metabolism in several brain areas, including bilateral temporal and parietal cortices (Figure 1a). MRI showed atrophy in bilateral parietal cortices, but the degree of atrophy was not marked in the hippocampus (Figure 1b). Patient took drugs for depression between 2000–2001, but she did not have depression at the time of admission with memory dysfunctions (2014). The *APOE* genotype of patient was homozygous for the E3 allele. No family members reportedly displayed any neurodegenerative phenotypes. All living relatives refused genetic testing or the release of their medical records.

The second case was a 49-year-old male, whose initial symptom was short-term memory dysfunction. According to the caregiver’s report, the patient had worked as a bus driver for more than 30 years but began to complain of difficulty operating the bus and keeping track of time. By 50 years of age, the patient often forgot the code to his front door. He quit his job a year later, after which his cognitive functions worsened to the point where he regularly lost items and was unable to use his mobile phone or TV remote control. His language function worsened, and he frequently repeated himself or repeated words. During this time, the patient visited another mental health institution and received treatment for panic disorder. His *APOE* genotype was E3/E3. His CDR and MMSE scores were 1.5/3 and 15/30, respectively. Amyloid PET showed amyloid positivity in several brain regions, including bilateral lateral temporal-frontal-parietal areas, posterior cingulate, and precuneus (Figure 1c). Brain MRI did not reveal any brain atrophy or any lesions in the brain parenchyma (Figure 1d). His mother was also diagnosed with dementia, but his two siblings did not develop any cognitive symptoms. All living relatives disagreed with the genetic testing or declined to provide any additional information on their condition.

This study involving human participants was reviewed and approved by Institutional Review Board of Seoul National University Bundang Hospital (B-1612/376-701, Patient 1) and by Gachon University Gil Medical Center (GBIRB2018-013, Patient 2.). Written consents were obtained from patients (or their caregivers), involved in this study.

### 2.2. Molecular Genetic Analysis

DNA was extracted from white blood cells by a GeneAll blood kit (Seoul, Korea) as described in the kit’s protocol. An extensive genetic screen was performed on proband, on 50 genes, verified as causative and risk factors for different neurodegenerative diseases [11,13]. Extensive genetic analysis for the 50 disease-causing and disease-risk genes was performed on the first patient by Theragen Etex Bio Institute (Seoul, Korea). For the second patient, whole exome sequencing (WES) was performed by Novogene, Inc. (https://en.novogene.com; Beijing, China), and patient was also screened for the same 50 genes. To verify the mutations, Sanger sequencing was also done [11,13] by BioNeer, Inc. (Dajeon, Korea). The discovered mutations were screened in different reference databases, including the 622 unaffected individuals in Korean Reference Genome Database (KRGDB, http://152.99.75.168/KRGDB/menuPages/intro.jsp), the Genome Aggregation Database (*gnomAD*, https://gnomad.broadinstitute.org/), and 1000 Genomes (http://www.1000genomes.org/) databases.

### 2.3. In Silico Analyses and Protein Structure Prediction

The mutation was screened by PolyPhen-2 (http://genetics.bwh.harvard.edu/pph2/), Sorting Intolerant From Tolerant algorithm (SIFT; http://sift.jcvi.org/), and PROVEAN (http://provean.jcvi.org/index.php) software, which estimate the nature possible damaging nature of mutations. Predictions on mutations were also done by Expasy server (https://www.expasy.org/) on different parameters, including bulkiness, polarity, or hydrophobicity (Kyte and Doolittle scale). Structures of mutant and normal proteins were modeled by Raptor X software (http://raptorx.uchicago.edu/), and by Discovery Studio 3.5 Visualizer software, designed by Accerlrys [14].

## 3. Results

### 3.1. Mutation Analysis

**Figure 2:**
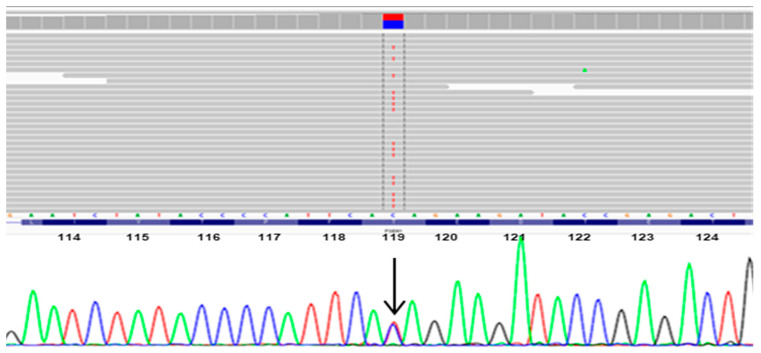
Whole exome sequencing (WES) data of the PSEN1 Thr119Ile (c.356C>T) mutation, verified by Sanger sequencing.

**Figure 3:**
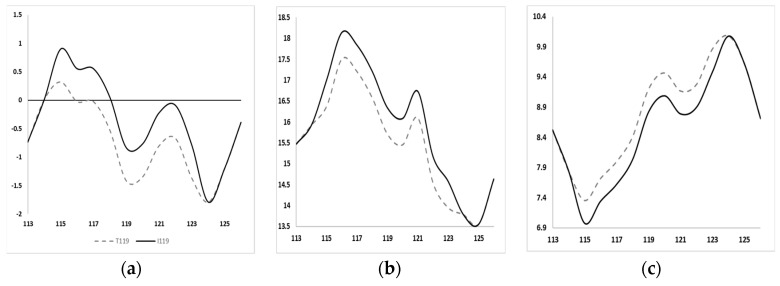
ExPasy predictions on *PSEN1* Thr119Ile. (**a**) Hydrophobicity scores increased due to the mutation, (**b**) bulkiness scores were higher due to isoleucine, and (**c**) polarity scores dropped in case of isoleucine 119.

A heterozygous variant c.356C>T was found in *PSEN1* codon 119, resulting in an exchange from threonine to isoleucine (Figure 2). *PSEN1* p.Thr119Ile is located in the hydrophilic loop (HL) region-I of the PSEN1 protein (Figure 3). *PSEN1* p.Thr119Ile did not appear in unaffected controls in reference databases (KRGDB, ExAC, or 1000 Genomes).

Genetic screening for the 50 genes revealed 24 and 31 variants in Patient 1 and Patient 2, respectively. In Patient 2, several rare variants appeared, which were missing in Patient 1, including ATP Binding Cassette Subfamily A Member 7 (*ABCA7)* Val1729Met, Sortilin Related Receptor 1 (*SORL1)* Asn828Ser, and Gly1524Arg. (Table 1).

### 3.2. In Silico Predictions

PolyPhen2 suggested Thr119Ile to be probable damaging with the score of 1.000 (HumDiv score). Multiple sequence alignment revealed that Thr119 may be conserved amongst vertebrates. In the PSEN-like sequences of different vertebrates (dogs, bats, or opossums), threonine was observed in the homologous position of Thr119. SIFT software scored this mutation as likely to be “tolerated” or probably nondamaging, with a score of 0.385.

ExPASY scores revealed significant changes due to the *PSEN1* Thr119Ile mutation. The hydrophobicity score was increased in the case of Ile119 (–0.833), compared to Thr119 (–1.411, Figure 3a). The bulkiness score was increased in the Thr119Ile mutation from 15.71 (Thr119) to 16.336 (Ile 119, Figure 3b). The mutation resulted in a reduction in the polarity score from 9.2 (Thr119) to 8.822 (Ile119, Figure 3c). *PSEN1* Thr119Ile may have also affected the hydrophobicity, bulkiness, and polarity scores of residues, located nearby (Tyr115-Glu123).

**Figure 4:**
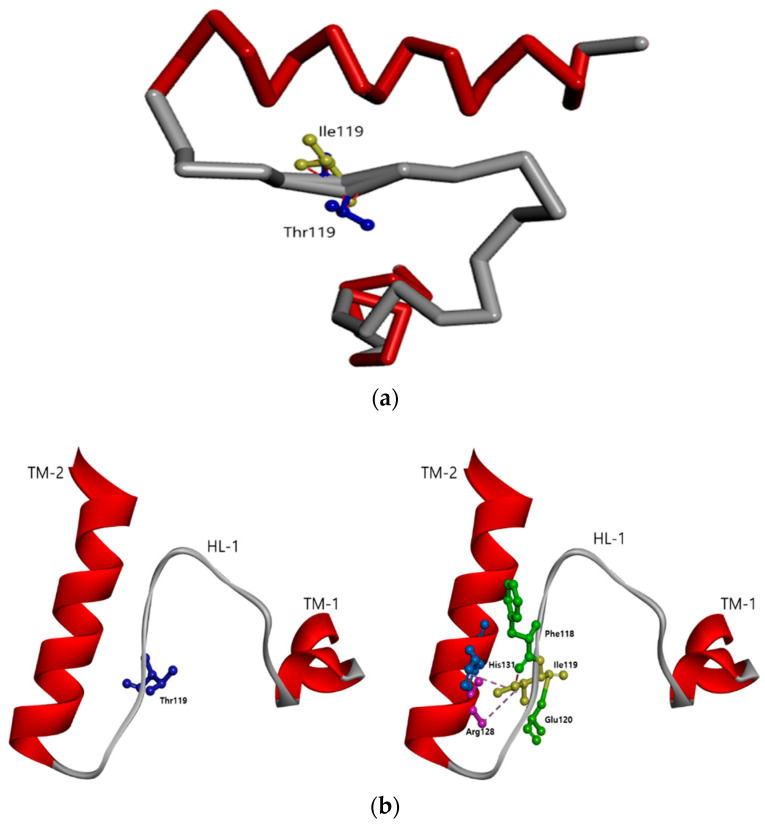
(**a**) Structure prediction of the *PSEN1* Thr119Ile mutation, compared to normal *PSEN1.* Mutation may result in significant disturbances in HL-I loop and may result in altered amyloid metabolism. (**b**) Intramolecular interactions in the case of the *PSEN1* Thr119Ile mutation, compared to normal *PSEN1.* The hydrophobic isoleucine may form additional intramolecular interactions with amino acids, located in TM-2.

Threonine is a hydrophilic residue with the ability to form hydrogen bonds because of its hydroxyl group. However, substitution with a nonpolar isoleucine amino acid may lead to changes in the protein’s intra- and intermolecular interactions. Hence, the Thr119Ile mutation was expected to significantly disturb the HL-1 region (Figure 4a). 3D modeling of the mutant PSEN1 protein also revealed abnormal orientation of loop, and mutation also changed the intramolecular interactions occurring within the PSEN1 protein. Although Thr119 may not directly interact with any of its neighboring amino acids, substitution with Ile119 may result in stronger interactions between the HL-1 loop and transmembrane helix-2 (TM-2). In addition, Ile119 may form hydrogen bonds with Glu120 and Phe118, in turn enhancing contact with TM-2 (Figure 4b).

## 4. Discussion

In the current study, we described a probable pathogenic *PSEN1* missense mutation (p.Thr119Ile) in two unrelated Korean patients, diagnosed with EOAD. *PSEN1* p.Thr119Ile was described by our group for the first time among Asian populations [11,12]. This mutation was previously described in a large family from Argentina (Table 2). The Argentinian proband patient developed memory impairment at the age of 49. MMSE and CDR tests revealed mild cognitive dysfunction, which progressed to severe dementia by his 70s. A cousin of the proband, who was also positive for the mutation, experienced memory impairment at 54 years of age. He developed AD when he was 71 years of age, and died 8 years later. Parental and grandparental generations were also severely affected with AD, which manifested in their 60s or early 70s. The Argentinian proband’s FDG-PET revealed hypometabolism in different brain regions, including the frontal, parietal, and lateral temporal areas. PiB-PET showed amyloid depositions in different brain areas, such as in the frontal, parietal, posterior cingulate, and lateral temporal regions. Cerebrospinal fluid (CSF) biomarker studies were inconsistent, as the proband patient had reduced Aβ42 in the CSF, while his cousin showed increased phospho-Tau levels [15]. This mutation was also found in a Chinese family, where proband was diagnosed with behavioral variant late onset AD. Personality changes, such as aggression, social withdrawal, or irritability, appeared in her late 60s. Her cognitive and executive functions were also disturbed, and her memory decline became more severe at the age of 72. She did not develop myoclonus, seizure, or ataxia. Family history was definitely positive, since her grandmother and her sisters also developed similar phenotypes. Age of onset was later in the Chinese family, compared to the Korean and Argentinian cases [16]. Cognitive dysfunction in the two unrelated Korean cases with identical mutations manifested at a similar age and had a similar clinical course, compared to the Argentinian cases. The Thr119Ile mutation was also associated with positive amyloid PET in at least three cases (Argentinian, Chinese, and Korean patient 2), suggesting that the mutation plays a role in AD pathology. No relation was found between these two Korean cases of *PSEN1* Thr119Ile, which suggested independent occurrences of two cases. Patient 1 seemed to be a de novo case of mutation, since no other family member with any cognitive dysfunction was found. Mother of Patient 2 developed dementia, suggesting a probable familial case of AD. *PSEN1* Thr119Ile was also reported in a Chinese family, but it would be difficult to find any connection. However, the possibility of common founder cannot be ruled out completely. Previously, the pathogenic *PSEN1* Thr116Ile was reported in two families with strong family history without any connection. In case of the families with Thr116Ile, the possibility of common founder could be higher, but it may be hard to prove [17].

Among the two patients, Patient 2 represented with earlier disease onset with rapid progressions of the disease, and additional genetic factors cannot be ruled out. Genetic analysis for the 50 genes for AD, FTD, PD, and other diseases revealed differences on the mutation pattern of Patient 1 and Patient 2 (Table 1). Patient 2 carried a novel mutation in *ABCA7* (Val1729Met) and 2 rare *SORL1* (Asn828Ser and Gly1524Arg) mutations. In addition, Patient 2 also carried a known common AD risk factor *SORL1* Arg528Thr [18]. The difference in rare mutation pattern may also support the independent occurrence in these two cases. In addition, it may be possible that rare variants in *SORL1* and *ABCA7* could act as genetic modifiers for the faster progressions of the disease with aggressive phenotype. Even though *ABCA7* and *SORL1* were also suggested as strong risk factors for LOAD, emerging evidence points to their role in EOAD [19,20,21]. *SORL1* mutations could play a role in slowing down the APP transport to cell surface and endosome system through the endoplasmic reticulum -Golgi system. This process could result in faster γ-and β -secretase cleavages [19]. *ABCA7* mutations could also contribute to EOAD risk by reducing the *ABCA7* expressions. *ABCA7* deficiency could also enhance the amyloid cleavage and APP proteolysis. In addition, *ABCA7* dysfunctions could reduce the amyloid clearance [21,22]. *ABCA7, SORL1*, and *PSEN1* may interact through amyloid metabolism [13]. These additional risk genes, such as *ABCA7* and *SORL1*, may accelerate the effects of *PSEN1* Thr119Ile.

The PSEN1 Thr119Ile mutation is located in the evolutionarily highly conserved area of PSEN1 protein, known HL-I loop, between TM-I and TM-II helices. Because of its location and interaction with neighboring amino acids, a Thr to Ile exchange results in abnormalities in PSEN1 functions, which could drive the disease pathology. We found that in silico analyses and structure modeling of PSEN1 predicted that this mutation could result in significant torsion of PSEN1 HL-I loop. PSEN1 Thr116Ile were reported in two Korean families, which may also disturb the HL-I loop significantly [17]. Both Thr116Ile and Thr119Ile would enhance the hydrophobic interactions of residues in HL-I, and may make HL-I less favorable for lumen region. Structure predictions on these mutations suggested that these variants may shift the HL-I towards to TM regions (either TM-1 or TM-2). Several other probable pathogenic mutations in this loop could cause early onset of AD, occurring in patients’ 40s–60s [23,24,25]. Younger onset AD cases, which occurred before 40 years or even 30 years of age, have also been reported (Pro117Leu, Pro117Ala) [26]. All patients who carried mutations in the HL-I loop presented with memory dysfunction, but other symptoms have also been described such as Parkinsonism (Phe105Leu) [21,22], myoclonic jerks (Arg105Gln; Tyr115His, n1998; Pro117Leu) [26,27,28,29,30], epileptic seizures [31], and spastic paraparesis (Glu120Lys) [32]. PSEN1 HL-I mutations have also been shown to play a significant role in γ-secretase activity, including the catalysis and endoprotolysis of its substrates and APP cleavage [33,34,35].

Threonine has a hydroxyl group, and has the ability to form hydrogen bonds, while isoleucine is a hydrophobic residue. In silico modeling revealed that Thr119Ile could contribute to considerable alterations in the HL-I loop structure. The Ile119 mutation may result in additional intramolecular interactions, which could enhance the proximity between HL-I and TM-II (Figure 4). Taken together, protein conformational changes caused by Thr119Ile could perturb the normal function of presenilin1 and γ-secretase, leading to the development of AD [33,34,35,36].

Limitations of this study include the inability to perform a segregation analyses of PSEN1 Thr119Ile in relatives of patients, since they did not agree the test. We also could not perform analysis of biomarkers, since no brain specimens, plasma, or CSF samples were available from patients. Additional functional in vivo and in vitro studies of the *PSEN1* Thr119Ile mutation will be needed to support its pathogenicity.

## 5. Conclusions

In conclusion, our findings suggest that *PSEN1* Thr119Ile may be a probable pathogenic mutation. This mutation has relatively wide range of age onset, suggesting that additional genetic factors, especially in AD risk genes, could contribute as disease modifiers, which may result in accelerated or delayed reduced progression. Even though we could not perform in vitro studies in cell models for the *PSEN1* Thr119Ile mutation, the conducted structure predictions combined with information from previous studies demonstrate the pathogenic nature of the Thr>Ile substitution in *PSEN1* HL-I region. Additionally, functional studies are needed to find out how this mutation could play a role in AD pathogenesis.

## Figures and Tables

**Table 1:** Mutation profile for the two patients with PSEN1 Thr119Ile. Rare variants in PSEN1, SORL1 and ABCA7 have been highlighted with bold.

Gene	Chromosome	Mutation	Patient 1	Patient 2	Rs ID	1000g	ExAC	SIFT	Polyphen2
*ABCA7*	19	E188G	found	found	rs3764645	0.399561	0.4838	0.647, T	0.358, B
		R463H	NA	found	rs3752233	0.060703	0.0478	0.254, T	0.997, D
		N718T	found	found	rs3752239	0.059105	0.0703	0.239, T	0.529, P
		G1527A	found	found	rs3752246	0.825479	0.8405	0.877, T	0.0, B
		Q1686R	found	found	rs4147918	0.05651	0.0479	0.234, T	0.001, B
		**V1729M**	**NA**	**found**	**NA**	**NA**	**NA**	**0.151, T**	**0.1, B**
		A2045S	found	found	rs4147934	0.605032	0.7317	0.962, T	0.057, B
*ALS2*	2	V368M	found	found	rs3219156	0.896565	0.9106	0.191, T	0.006, B
*BACE1*	11	C412R	found	found	rs539765	1	0.9997	1.0, T	0.0, B
*BIN1*	2	R263Q	NA	found	rs117721706	0.004593	0.0015	0.349, T	0.997, D
*CR1*	1	T1858M	found	found	rs3737002	0.248802	0.275	0.021, D	1.0, D
		T2060S	found	found	rs4844609	0.995008	0.9853	1.0, T	0.003, B
		T2419A	found	found	rs2296160	0.828075	0.8159	1.0, T	0.0, B
*CTNNA3*	10	S596N	found	found	rs4548513	0.485024	0.412	1.0, T	0.0, B
*LRP6*	12	V1062I	found	found	rs2302685	0.885583	0.8474	1.0, T	0.0, B
*LRRK2*	12	R50H	found	found	rs2256408	0.969249	0.9911	1.0, T	0.0, B
		N551K	found	found	rs7308720	0.099441	0.0861	0.009, D	1.0, D
		R1398H	found	found	rs7133914	0.100439	0.0841	0.1, T	0.992, D
		M2397T	found	found	rs3761863	0.551717	0.624	0.466, T	0.0, B
*MAPT*	17	Y441H	found	found	rs2258689	0.312899	0.2752	0.978, T	0.001, B
*NOTCH3*	19	A2223V	found	found	rs1044009	0.629393	0.7591	0.175, T	0.001, B
*OPTN*	10	K322E	found	found	rs523747	0.993411	0.9973	1.0, T	0.0, B
*PARK2*	6	S167N	NA	found	rs1801474	0.117412	0.0676	0.229, T	0.027, B
*PSEN1*	**14**	**T119I**	**found**	**found**	**NA**	**NA**	**NA**	**0.385, T**	**0.998, D**
*SIGMAR1*	9	Q2P	found	found	rs1800866	0.217252	0.184	0.513, T	0.0, B
*SORL1*	11	A528T	NA	found	rs2298813	0.103235	0.0722	0.306, T	0.962, D
		**N828S**	**NA**	**found**	**rs377222446**	**0.0002**	**0.0002**	**0.745, T**	**0.0, B**
		Q1074E	found	found	rs1699107	0.984824	0.9949	1.0, T	0.0, B
		**G1524R**	**NA**	**found**	**rs201415809**	**0.0008**	**0.0002**	**0.008, D**	**1.0, D**
		V1967I	found	found	rs1792120	0.979433	0.9953	1.0, T	0.0, B
*SPG11*	15	F463S	found	found	rs3759871	0.47484	0.4659	0.343, T	0.066, B

**Table 2:** Clinical phenotypes of the *PSEN1* Thr119Ile missense mutation of two Korean cases and cases from Argentina.

	Argentinian Family	Chinese Family	Korean-1	Korean-2
Age of onset (years)	49–71 years	Late 60s	64 years	49 years
Family history	Positive	Positive	Probably *de novo*	Positive
Disease	EOAD	LOAD, behavioral variant	EOAD	EOAD
CDR	0.5, later 3	2	0.5	1.5
MMSE	28/30	16/30	28/30	14/30
Amyloid PET	Positive, frontal, parietal, precuneus/posterior cingulate, lateral temporal, and striatum	Diffuse amyloid retention in bilateral-parietal and temporal cortex	NA	Positive, posterior cingulate, and precuneus
FDG-PET	Mild bilateral hypometabolism in parietal lobe, precuneus, anterior cingulate, dorsal frontal lobe, and lateral temporal lobe with left predominance	NA	Bilateral hypometabolism in parietal and temporal cortices	NA
MRI	NA	Atrophy in frontal-temporal lobe, shrinkage of hippocampus	Global atrophy	No significant changes
CSF biomarkers	Reduced Aβ or elevated Tau	NA	NA	NA
References	[15]	[16]	[11,12] recent finding	Recent finding
